# Tailoring ordered structures with distorted [TeO_3_] and aligned [ScO_6_] motifs for balanced nonlinear optical properties in rare-earth tellurate crystals

**DOI:** 10.1039/d5sc07486j

**Published:** 2025-11-18

**Authors:** Xiaoxu Wang, Tinghui Zhang, Huijian Zhao, Ning Jia, Hongjun Liu, Ning Ye, Zhanggui Hu, Conggang Li

**Affiliations:** a State Key Laboratory of Crystal Materials, Tianjin Key Laboratory of Functional Crystal Materials, Institute of Functional Crystal, Tianjin University of Technology Tianjin 300384 China cgli@email.tjut.edu.cn; b Institute of Optics and Fine Mechanics, Chinese Academy of Sciences Shanghai 201800 China jianing@siom.ac.cn; c Shanghai Key Laboratory of Wide and Ultra-Wide Bandgap Semiconductor Materials Shanghai 201800 China

## Abstract

Tailoring nonlinear optical (NLO) crystals with a large bandgap and wide transparency extending from ultraviolet (UV) to mid-infrared (IR) wavelengths remains a challenge due to the inherent trade-offs in these properties. Here, we report two novel scandium tellurite NLO crystals, AScTe_2_O_6_ (A = K, Rb), engineered through a synergistic strategy that optimizes both electron distribution and lattice vibrations. These isostructural crystals feature a unique 2D topological framework analogous to KBBF, built from distorted [TeO_3_] pyramids and [ScO_6_] octahedra. Notably, RbScTe_2_O_6_ achieves an ultrawide bandgap of 4.43 eV with a UV cutoff at 236 nm, the shortest reported among known NLO tellurates, coupled with an extended IR transparency beyond 7.0 µm. Moreover, it affords a compelling combination of a high laser-induced damage threshold of 27.8 × AgGaS_2_, a strong phase-matchable second harmonic generation response of 2.3 × KDP, and a large birefringence of 0.19 at 1064 nm. The highly polarizable [TeO_3_] pyramids and well-aligned [ScO_6_] octahedra collectively dominate the optical anisotropy and NLO activity, as evidenced by first-principles calculations and dipole moment analyses. These outstanding attributes establish RbScTe_2_O_6_ as a highly promising candidate for NLO applications in wide wavelength ranges.

## Abstract 2

Tailoring nonlinear optical (NLO) crystals with a large bandgap and wide transparency extending from ultraviolet (UV) to mid-infrared (IR) wavelengths remains a challenge due to the inherent trade-offs in these properties.

## Graphical abstract

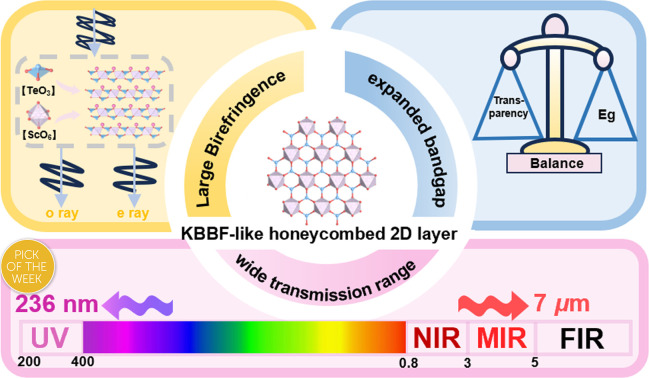

## Introduction

The exploration and fabrication of inorganic oxides with remarkable optoelectronic properties have garnered considerable attention.^1–4^ Among these, non-centrosymmetric (NCS) structures are of paramount significance due to their functional characteristics such as piezoelectricity, ferroelectricity, pyroelectricity, and nonlinear optical (NLO) frequency conversion capability.^5–10^ Significant progress has been made in advancing high-performance oxide-based NLO crystals, exemplified by borates such as KBe_2_BO_3_F_2_ (KBBF),^11^ β-BaB_2_O_4_ (β-BBO),^12^ LiB_3_O_5_ (LBO),^13^ and CsLiB_6_O_10_ (CLBO) crystals,^14^ as well as phosphates such as KH_2_PO_4_ (KDP) and KTiOPO_4_ (KTP).^15,16^ Although these materials have found extensive applications from ultraviolet (UV) to the near-infrared (IR) wavelengths, their mid-IR transparency typically remains confined to wavelengths below 4.5 µm, primarily due to the high phonon energies associated with light elements such as boron and phosphorus.^17^ Consequently, the pursuit of oxide-based NLO materials capable of extending mid-IR transparency represents a critical research direction in laser science and technology.^18^

Recent studies have demonstrated that a prevalent strategy for designing NLO crystals with broad IR transparency involves the incorporation of NLO-active structural chromophores, including transition metal cations with *d*^0^ electronic configuration (*d*^0^-TM, *e.g.*, Ti^4+^, Zr^4+^, W^6+^, and Mo^6+^) and stereo-chemically active lone pair cations (SCALPs, *e.g.*, Pb^2+^, Bi^3+^, Te^4+^, and I^5+^).^19–22^ These functional units are susceptible to generating acentric configurations and enhancing second harmonic generation (SHG) responses. Moreover, the incorporation of heavy elements (*e.g.*, Pb, Bi, and Te) characterized by low phonon energy facilitates a broad transparency window extending well into the mid-IR region. Notable examples include BaTeM_2_O_9_ (M = Mo, W),^23^ A_2_BiVO_6_ (A = Cd, Ca),^24,25^ and Na_0.5_Bi_2.5_Nb_2_O_9_,^26^ showcasing a wide mid-IR transparency range. Nevertheless, the introduction of *d*^0^ transition metals typically leads to a reduction in the bandgap (<3.0 eV), resulting in a red-shifted UV absorption edge.^27^ Thus, the simultaneous achievement of a broad transparency from the UV to mid-IR wavelengths and a large bandgap remains a key challenge.^28^

In contrast to transition metals, rare-earth cations offer versatile structural adaptability and can facilitate NCS coordination environments.^29^ Additionally, closed-shell rare-earth ions such as Sc^3+^, Y^3+^, and La^3+^ lack *d–d* or *f–f* electronic transitions, thereby enabling the attainment of large bandgaps.^30–33^ It is thus proposed that in oxide systems incorporating both *d*^0^ transition metals and SCALPs, substitution of the *d*^0^ metal cation with rare-earth ions could effectively enlarge the bandgap; concurrently, retention of heavy SCALP elements with low phonon energy could inherit a wide mid-IR transmission range. This dual approach, incorporating considerations of electronic configurations and phonon characteristics, presents a promising avenue for balancing a wide bandgap and expansive optical transparency.

Guided by these insights, using the chemical substitution method,^34^ two novel rare metal scandium tellurate NLO crystals, namely AScTe_2_O_6_ (A = K, Rb) (KSTO and RSTO), were identified by integrating Sc^3+^ ions with tellurate groups, achieving synergistic optimization of electron distribution and lattice vibrations. These isostructural compounds adopt a KBBF-like quasi-two-dimensional (2D) architecture composed of [TeO_3_] pyramids and [ScO_6_] octahedra. Notably, RSTO demonstrates an enlarged bandgap of 4.43 eV, revealing a short UV cutoff edge at 236 nm, exceeding most known tellurates, and an extended transmission window reaching to 7.0 µm, which fully covers the 3–5 µm atmospheric transmission windows. In addition, RSTO demonstrates a high laser damage threshold (LDT) of 27.8 × AgGaS_2_ and a strong phase-matchable SHG response of 2.3 times that of KDP, alongside a large birefringence of 0.19 at 1064 nm. These outstanding properties establish RSTO as a highly promising candidate for NLO applications spanning from UV to mid-IR wavelengths. The proposed synergetic strategy integrating electron distribution and phonon engineering offers a novel avenue for tailoring high-performance NLO materials with a wide bandgap and broad transparency range.

## Experimental section

### Synthesis and powder X-ray diffraction (PXRD)

Polycrystalline samples of RSTO were synthesized by a high temperature solid–state reaction. Stoichiometric amounts of Rb_2_CO_3_ (99.99% purity), Sc_2_O_3_ (99.99% purity), and TeO_2_ (99.99% purity) were thoroughly ground, placed into an alumina crucible, and preheated at 300 °C for 24 h. The resulting powder were further ground, pressed into pellets, sealed in evacuated quartz tubes, and sintered at 600 °C for 96 h, followed by slow cooling to room temperature over 48 h. Phase purity was confirmed by PXRD using a Rigaku diffractometer (SmartLab, 9 KW) with Cu Kα radiation (*λ* = 1.54186 Å) in the 10–70° range with a step size of 0.01°.

### Single-crystal preparation

Single crystals of AScTe_2_O_6_ (A = K, Rb) were grown through a mild hydrothermal method. For KSTO, a mixture of K_2_CO_3_ (0.414 g, 3.00 mmol), Sc(NO_3_)_3_·6H_2_O (0.339 g, 1.00 mmol), TeO_2_ (0.478 g, 3.00 mmol), and 5 mL deionized water was sealed in a 23 mL Teflon-lined autoclave. For RSTO, Rb_2_CO_3_ (0.554 g, 2.40 mmol), Sc(NO_3_)_3_·6H_2_O (0.277 g, 0.82 mmol), TeO_2_ (0.383 g, 2.40 mmol), and 5 mL deionized water were used. The reactors were heated at 230 °C for 4 days and were cooled to room temperature at 3–6 °C h^−1^. The reaction products were recovered by filtration and washed several times with water, and then dried at room temperature. Finally, colorless crystals were obtained (Fig. S1, SI).

### Single-crystal XRD measurement

Single crystals of the title compounds were subjected to X-ray diffraction analysis on a Bruker APEX III CCD diffractometer with Mo Kα radiation (*λ* = 0.71073 Å) at 298 K. The collected data underwent integration and absorption correction processes utilizing SAINT software.^35^ Subsequently, the crystal structure was elucidated through intrinsic phasing (SHELXT) and refined *via* full-matrix least-squares fitting on *F*^2^ (SHELXL), with anisotropic modeling of all non-hydrogen atoms. The validity of the structural arrangement and symmetry was confirmed through analysis with PLATON software.^36^

### Energy-dispersive spectroscopy

Elemental composition and homogeneity of RSTO crystals was analyzed using energy-dispersive X-ray spectroscopy (EDS) on an FEI Quanta FEG 250 field-emission scanning electron microscope.

#### Optical spectrum measurements

Ultraviolet-visible-near infrared (UV-Vis-NIR) diffuse reflectance spectra were collected at room temperature on a Hitachi UH4150 spectrophotometer over the range of 200–1900 nm. The experimental band gaps of RSTO can be determined based on the Kubelka–Munk formula.^37^ The infrared (IR) spectra of RSTO were recorded within the wavenumber range of 400–4000 cm^−1^ using a Nicolet iS50 FT-IR IR spectrometer.

### Second harmonic generation (SHG) evaluation

The SHG response of RSTO polycrystalline samples were evaluated using a Q-switched Nd:YAG laser with a wavelength of 1064 nm *via* the Kurtz–Perry method.^38^ RSTO samples were sieved into distinct particle size ranges: 53–75, 75–106, 106–120, 120–150, 150–180, and 180–212 µm. Powdered KDP was used as a reference under identical conditions.

### LIDT measurement

The laser-induced damage threshold (LIDT) measurement of RSTO was performed using a Q-switched pulsed laser (1064 nm, 10 ns pulse width, 1 Hz). The particle size range of the tested samples is 150–210 µm with the AgGaS_2_ (AGS) sample comparable particle size. Laser fluence was controlled *via* a variable attenuator, and the spot size was calibrated by adjusting the sample-to-laser distance. The LIDT value was determined using an energy ramping technique, wherein the laser energy output was progressively increased until visible surface damage was observed. The LIDT value was calculated using the following equation: LIDT = *E*/(*s* × *t*), where *E* denotes the recorded laser energy, *s* represents the damaged area, and *t* signifies the pulse duration. The measured damage areas for RSTO and AGS were 9.67 × 10^−3^ and 0.062 cm^−2^, respectively, with corresponding damage energies of 15.3 and 3.5 mJ, yielding LIDT values of 158 and 5.68 MW cm^−2^, respectively.

### Birefringence characterization

The birefringence of RSTO was characterized using the polarizing microscope (Nikon Eclipse E200MV) equipped with a Berek compensator. The retardation *R* was measured, and birefringence Δ*n* was calculated *via* the formula *R* = |*N*_g_ − *N*_p_| × *d* = Δ*n* × *d*, where *N*_g_ and *N*_p_ are refractive indices of the fast and slow rays, respectively, and *d* is crystal thickness. The thickness of the selected RSTO crystal was ascertained using a Bruker SMART APEX III CCD diffractometer.

### Theoretical calculation

First-principles calculations of RSTO based on density functional theory (DFT) were performed using the CASTEP package.^39^ The Perdew–Burke–Ernzerhof (PBE) generalization and generalized gradient approximation (GGA) functional were applied for all calculations.^40^ A kinetic energy cutoff of 810 eV and a Monkhorst–Pack *k*-point mesh of 3 × 3 × 2 were used to ensure convergence. Electronic structures and optical properties were analyzed to interpret the experimental findings.

## Results and discussion

### Synthesis and phase characterization

Polycrystalline samples of RSTO were prepared by a high temperature solid–state reaction in vacuum quartz tubes. Analysis of the powder X-ray diffraction (PXRD) patterns, as shown in Fig. S2, confirmed the high purity of the synthesized product RSTO, with experimental curves exhibiting excellent alignment with the proposed structural model.

### Crystal structure determination

**Fig. 1:**
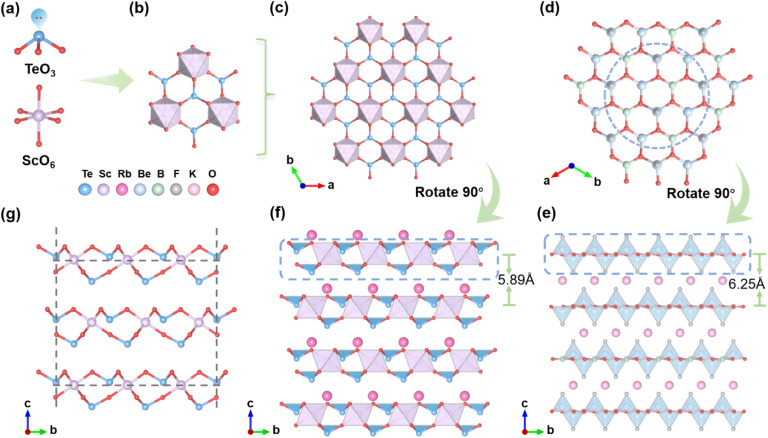
Structural features of RSTO and KBBF. (a) Basic building units: [TeO_3_] pyramids and [ScO_6_] octahedra. (b) A [Te_4_Sc_3_O_21_] cluster formed by interlinked [TeO_3_] and [ScO_6_] units. (c and d) Comparison of the quasi-2D [Te_4_Sc_3_O_21_]_∞_ honeycomb layers in RSTO and 2D [Be_2_BO_3_F_2_]_∞_ layers in KBBF. (e and f) Stacking patterns of [Be_2_BO_3_F_2_]_∞_ and [Te_4_Sc_3_O_21_]_∞_ layers in KBBF and RSTO, respectively. (g) Spatial alignment of structure composed of [TeO_3_] and [ScO_6_] units viewed along the bc plane.

Single-crystal XRD analysis reveals that both RSTO and KSTO crystallize in the hexagonal space group *P*6_3_*mc* (no. 186). The unit cell parameters for RSTO are *a* = *b* = 5.8371 (3) Å, *c* = 11.7832 (13) Å, and *V* = 347.69 (5) Å^3^, and for KSTO, *a* = *b* = 5.7988 (3) Å, *c* = 11.5416 (10) Å, and *V* = 336.10 (5) Å^3^. Detailed crystallographic data are presented in Tables 1 and S1–S4. Given their structural similarity, RSTO is selected as a representative for detailed description. Within the unit cell, there are two distinct Te atoms, one Rb atom, one Sc atom, and two O atoms. As shown in Fig. 1a, the Sc atoms are bonded to six O atoms, forming a distorted [ScO_6_] octahedron with Sc–O bond lengths in the range of 2.089 (6) to 2.115 (6) Å. The two crystallographically independent Te atoms are coordinated by three oxygen atoms, forming [TeO_3_] triangular pyramids with stereo-chemically active lone pairs (SCALPs). The presence of SCALPs on Te^4+^ induces significant local distortion in the [TeO_3_] units, with Te–O bond lengths between 1.851 (6) and 1.875 (6) Å. Four [TeO_3_] triangular pyramids and three [ScO_6_] octahedra are further interconnected *via* corner-sharing oxygen atoms, forming [Te_4_Sc_3_O_21_] clusters that extend within the ab plane to form a honeycomb-like quasi- 2D layer (Fig. 1b and c). It is worth noting that this honeycomb-like 2D layer is analogous to the benchmark KBBF family with similar 2D [Be_2_BO_3_F_2_]_∞_ layers (Fig. 1d). Specifically, the [BeO_3_F] tetrahedra and [BO_3_] groups in KBBF are functionally replaced by [TeO_3_] pyramids and [ScO_6_] octahedra in RSTO. As demonstrated in Fig. 1e and f, these [Be_2_BO_3_F_2_]_∞_ layers and [Te_4_Sc_3_O_21_]_∞_ layers are stacked along the *c*-axis to form a framework with alkali metal ions K^+^ in KBBF and Rb^+^ in RSTO. Notably, the interlayer spacing in RSTO was found to be 5.89 Å, significantly smaller than that observed in KBBF (6.25 Å), suggesting improved suppression of layer-oriented growth tendencies. As demonstrated in Fig. 1g, the nearly orderly arrangement of the highly polar [TeO_3_] pyramids and [ScO_6_] octahedra contributes markedly to the macroscopic polarization anisotropy and NLO response. Bond valence sum (BVS) calculations performed on RSTO indicate the average valence states 3.03 for Sc, 4.09 for Te, 0.97 for Rb, and 1.97 for O, in good agreement with theoretical expectations.^41^ Moreover, energy-dispersive X-ray spectroscopy (EDS) analysis further supports the stoichiometry, yielding an average molar ratio of Rb : Sc : Te : O of 1.00 : 1.15 : 2.42 : 6.30, consistent with the theoretical composition of RSTO (Fig. S3).

**Table 1:** Crystal data and structure refinement for AScTe_2_O_6_ (A = K, Rb)

Empirical formula	KScTe_2_O_6_	RbScTe_2_O_6_
Formula weight	435.27	481.64
Temperature (K)	293 (2) K	293 (2) K
Wavelength (Å)	0.71073	0.71073
Crystal system	Hexagonal	Hexagonal
Space group	*P*6_3_*mc*	*P*6_3_*mc*
*a* (Å)	5.7988 (3)	5.8371 (3)
*b* (Å)	5.7988 (3)	5.8371 (3)
*c* (Å)	11.5416 (10)	11.7832 (13)
*V* (Å^3^)	336.10 (5)	347.69 (5)
Z	3	3
Density (g cm^3^)	4.301	4.601
*R*(int)	0.0378	0.0380
Completeness	99.3%	100.0%
GOF (*F*^2^)	1.044	1.203
*R*_1_, *wR*_2_ (*I* > 2σ(*I*))	*R*_1_ = 0.0202 *wR*_2_ = 0.0451	*R*_1_ = 0.0135 *wR*_2_ = 0.0354
*R*_1_, *wR*_2_ (all data)	*R*_1_ = 0.0253 *wR*_2_ = 0.0470	*R*_1_ = 0.0152 *wR*_2_ = 0.0359
CCDC number	2466750	2489720
*R*_1_ = ∑||*F*_o_| − |*F*_c_||/∑|*F*_o_|; *wR*_2_ = [∑*w* (*F*_o_^2^ − *F*_c_^2^)^2^/∑*w* (*F*_o_^2^)^2^]^1/2^		

### Spectroscopic properties

**Fig. 2:**
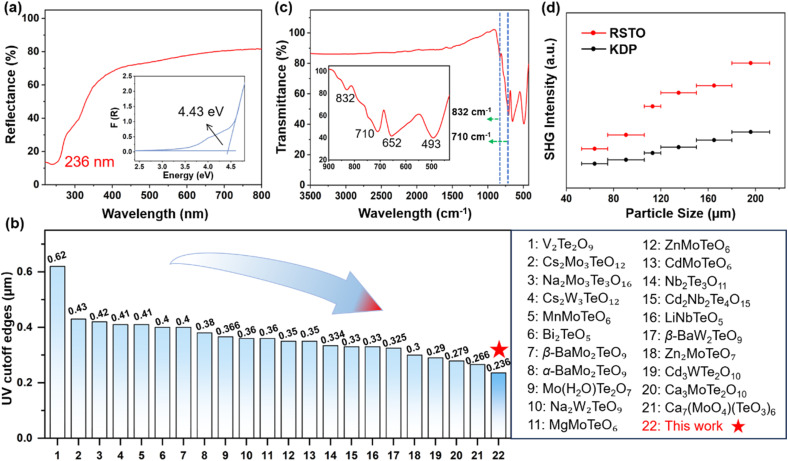
(a) UV-vis-NIR diffuse reflectance observation with the enlarged bandgap for RSTO. (b) Comparison of UV cutoff edges between RSTO and other representative NLO tellurate crystals. (c) IR spectrum for RSTO. (d) Phase-matching behavior evaluated by particle size-dependent SHG intensity, with KDP as a reference.

The UV-vis-NIR diffuse reflectance spectrum of RSTO was recorded over the range of 200–1900 nm. Notably, the analysis in Fig. 2a reveals a distinctive short UV cutoff edge at 236 nm, representing the shortest value reported among known NLO tellurates. This observation is further evidenced by a noticeable blue shift in comparison to other documented NLO tellurates (Fig. 2b and Table S5), such as β-BaMo_2_TeO_9_ (UV_cutoff_ = 400 nm),^42^ V_2_Te_2_O_9_ (UV_cutoff_ = 620 nm),^43^ Cs_2_Mo_3_TeO_12_ (UV_cutoff_ = 430 nm),^44^ and CdMoTeO_6_ (UV_cutoff_ = 350 nm).^45^ The corresponding optical band gap estimated from the absorption onset was determined to be 4.43 eV, surpassing those of commercial mid-IR NLO crystals including AGS (2.52 eV) and ZnGeP_2_ (1.75 eV),^46,47^ as well as the majority of transition metal tellurates. This wide band gap correlates with a high LIDT, measured to be 27.8 times that of AGS (Fig. S4), highlighting its potential for high-power laser applications. Infrared spectroscopy performed between 3500 and 500 cm^−1^ revealed a distinctive absorption peak at approximately 710 cm^−1^ and a weak feature around 832 cm^−1^ (Fig. 2c). The absorption peaks identified between 652 and 832 cm^−1^ can be attributed to the stretching vibrations of Te–O bonds,^48^ whereas the peaks around 493 cm^−1^ are assigned to the Sc–O vibrations. Notably, through two-photon absorption evaluation, RSTO exhibits a broad mid-IR transparency window extending to 6.0 µm (corresponding to 832 cm^−1^) with an IR absorption edge reaching 7.0 µm (corresponding to 710 cm^−1^), effectively covering the important 3–5 µm atmospheric window. These findings are in alignment with the comparative assessment involving tellurite compounds such as Pb_4_Ti_3_TeO_13_, Li_2_ZrTeO_6_, and Li_2_TiTeO_6_.^49–51^ In comparison to established oxide NLO crystals such as LiNbO_3_ (0.4–5 µm),^52^ KTP (0.35–4.5 µm),^16^ and RbTiOAsO_4_ (0.35–5.2 µm),^53^ RSTO showcases a significantly broader transparency range from short-wave UV to mid-IR wavelengths.

### SHG characterization

The SHG response of RSTO was evaluated using a Q-switched Nd:YAG laser at 1064 nm, with powdered KDP as a reference. As demonstrated in Fig. 2d, the SHG intensity increases proportionally with the gradual expansion of the particle size range and then reach the saturation level, which aligns with the expected phase-matching behavior as determined by the Kurtz–Perry method. Notably, within the particle size range of 180–212 µm, RSTO exhibits a strong SHG efficiency of 2.3 times that of KDP. Coupled with its wide band gap and extended transparency, these results affirm RSTO as a highly promising candidate for NLO applications across UV to mid-IR wavelength regions.

#### Birefringence characterization

**Fig. 3:**
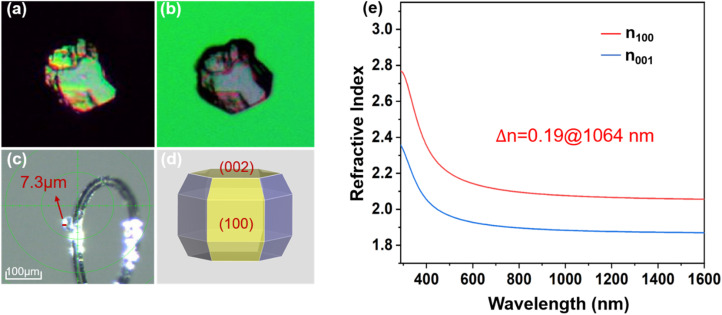
Birefringence characterization of RSTO. Optical micrographs under cross-polarized light showing (a) the interference color pattern and (b) the crystal at the extinction position. (c) Thickness measurement of the single-crystal sample. (d) Theoretical morphology of RSTO crystals. (e) Calculated refractive index dispersion curves derived from DFT-based simulations.

The birefringence of RSTO was experimentally determined using an interference color-based method. As shown in Fig. 3a–c, utilizing a crystal sample with a thickness of 7.3 µm, an optical path difference of 1375 nm was observed based on the Michel-Levy chart, yielding a measured birefringence value of 0.188 in the visible region. As illustrated in Fig. 3d, the theoretical crystal morphology of RSTO predicted by the BFDH method aligns well with its crystal structure. Moreover, refractive index dispersion curves were computationally derived (Fig. 3e and S5), confirming the uniaxial nature of RSTO and revealing a calculated birefringence of 0.19 at 1064 nm (compared to 0.17 for KSTO), consistent with the experimental observations. The obtained birefringence is comparable to those of widely used commercial birefringent crystals such as CaCO_3_ (0.172@589 nm) and YVO_4_ (0.223@630 nm),^54^ indicating that RSTO has large optical anisotropy for supporting phase-matching conditions. This notable birefringence primarily originates from the highly polarizable [TeO_3_] trigonal pyramids, where stereo-chemically active lone-pair electrons induce significant polarization anisotropy. Furthermore, the 2D layered structure of RSTO further amplifies the macroscopic structural anisotropy, collectively contributing to its large birefringent response.

### Structure–property correlations

**Fig. 4:**
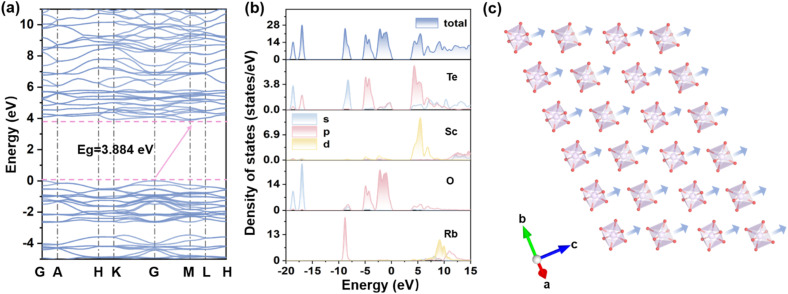
Structure–property correlation in RSTO. (a) Calculated band structure. (b) TDOS and PDOS plots. (c) Ordered alignment of [ScO_6_] octahedra with a uniform polarization orientation (blue arrow).

To gain insight into the origin of the optical properties of RSTO, density functional theory (DFT) calculations were carried out to analyze its electronic structure. As shown in Fig. 4a and S6, the band structure reveals that RSTO is an indirect bandgap material with a calculated bandgap value of 3.88 eV (compared to 3.334 eV for KSTO), smaller than the experimental result attributed to the known limitation of standard DFT in describing exchange-correlation energy discontinuities. Total and partial density of states (TDOS/PDOS) analyses indicate that the conduction band minimum (CBM) is dominated by Te 5p and Sc 3d orbitals, with a minor contribution from Te 5s states (Fig. 4b).

The valence band maximum (VBM) consists primarily of hybridized Te 5p and O 2p orbitals, suggesting strong covalent interactions in the Te–O bonds. Given the dominant contribution of these states near the Fermi level, the [TeO_3_] and [ScO_6_] units are identified as the primary sources of the linear and NLO responses in RSTO. Although the contribution from individual [ScO_6_] units is small, their ordered alignment cooperatively enhances the overall macroscopic polarization and optical anisotropy (Fig. 4c). According to these analyses, it can be concluded that both the highly polar [TeO_3_] pyramids and the systematically oriented [ScO_6_] octahedra play critical roles in the outstanding linear and NLO activities of RSTO.

## Conclusions

In summary, two novel scandium tellurite NLO crystals, RSTO and KSTO, were identified through a synergistic strategy of electron distribution modulation and phonon engineering. These compounds feature a KBBF-type 2D honeycomb layered structure composed of [TeO_3_] pyramids and [ScO_6_] octahedra. Notably, RSTO achieves an enlarged bandgap of 4.43 eV and an extended transparency window from the UV (236 nm) to the mid-IR cutoff beyond 7.0 µm, wider than most known NCS oxides. In addition, RSTO exhibits well-balanced combination of properties essential for practical NLO applications, including a high LIDT of 27.8 × AGS, a strong phase-matchable SHG response of 2.3 × KDP, and a sufficient birefringence of 0.19@1064 nm. These excellent properties position RSTO as a highly promising candidate for NLO applications across UV to mid-IR wavelength regions. The synergetic strategy integrating electron distribution and phonon engineering provides a new avenue for tailoring high-performing NLO materials that simultaneously exhibit wide bandgaps and broad transparency.

## Author contributions

Xiaoxu Wang: experiment, investigation, data curation, writing original draft. Tinghui Zhang and Huijian Zhao: experiment, software, formal analysis. Ning Jia: methodology, review & editing, funding acquisition. Hongjun Liu, Ning Ye, and Zhanggui Hu: resources, funding acquisition. Conggang Li: conceptualization, funding acquisition, methodology, project administration, review & editing.

## Conflicts of interest

There are no conflicts to declare.

## Supplementary Material

SC-017-D5SC07486J-s001

SC-017-D5SC07486J-s002

## Data Availability

The data supporting this article have been included as part of the supplementary information (SI). Supplementary information: crystallographic information, PXRD curves, EDS data, comparison of optical properties between selected NLO tellurates, LIDT values, calculated refractive index dispersion curves and Calculated band structure of KScTe_2_O_6_. See DOI: https://doi.org/10.1039/d5sc07486j. CCDC 2466750 (KScTe_2_O_6_) and 2489720 (RbScTe_2_O_6_) contain the supplementary crystallographic data for this paper.^55*a*,*b*^
