# Supplementary material for: Tailoring ordered structures with distorted [TeO3] and aligned [ScO6] motifs for balanced nonlinear optical properties in rare-earth tellurate crystals
Source: Chem Sci. 2025 Nov 18;17(5):2566–73. doi: 10.1039/d5sc07486j (PMC12690485; doi:10.1039/d5sc07486j)
Supplement: SC-017-D5SC07486J-s001 [file SC-017-D5SC07486J-s001.pdf]

Supplementary Information (SI) for Chemical Science.
This journal is © The Royal Society of Chemistry 2025

Electronic Supporting Information (ESI)

Tailoring ordered structures with distorted [TeO_3] and aligned [ScO_6] motifs for balanced nonlinear optical properties in rare-earth tellurate crystal

Xiaoxu Wang,[a] Tinghui Zhang,[a] Huijian Zhao,[a] Ning Jia,*[,b,c] Hongjun Liu,[a] Ning Ye,[a] Zhanggui Hu,[a] and Conggang Li*[,a]

[a]State Key Laboratory of Crystal Materials, Tianjin Key Laboratory of Functional Crystal Materials, Institute of Functional Crystal, Tianjin University of Technology, Tianjin 300384, China.

[b]Institute of Optics and Fine Mechanics, Chinese Academy of Sciences, Shanghai, 201800, China.

[c]Shanghai·Key·Laboratory of Wide·and Ultra-Wide Bandgap Semiconductor Materials, Shanghai, 201800, China.

***Corresponding author**, E-mail address: jianing@siom.ac.cn; cgli@email.tjut.edu.cn

1. **Table S1** Selected bond distances (Å) and angles (deg) for KSTO.

2. **Table S2** Selected bond distances (Å) and angles (deg) for RSTO.

3. **Table S3** Atomic coordinates (× 10^4), equivalent isotropic displacement parameters ($Å^2$ × 10^3), and the bond-valence sum for KSTO.

4. **Table S4** Atomic coordinates (× 10^4), equivalent isotropic displacement parameters ($Å^2$ × 10^3), and the bond-valence sum for RSTO.

5. **Table S5** Comparison of UV cutoff edges between RSTO and other representative NLO tellurate crystals.

6. **Fig. S1** Crystal morphology of (a) RSTO and (b) KSTO.

7. **Fig. S2** Experimental and calculated PXRD patterns of RSTO.

8. **Fig. S3** EDS analysis of RSTO.

9. **Fig. S4** Comparison of the LIDT values for AGS and RSTO.

10. **Fig. S5** Calculated refractive index dispersion curves of KSTO derived from DFT-based simulations.

11. **Fig. S6** Calculated band structure of KSTO.

Table S1 Selected bond distances (Å) and angles (deg) for KSTO.

Bond	Angle	Bond	Angle
Te(01)-O(1)#1	1.849(10)	O(1)-Sc(03)-O(005)	89.0(3)
Te(01)-O(1)#2	1.849(10)	O(005)#12-Sc(03)-O(005)	90.0(3)
Te(01)-O(1)#3	1.849(10)	O(005)#13-Sc(03)-O(005)	90.0(3)
Te(02)-O(005)#7	1.878(7)	O(005)#14-K(004)-O(005)#15	59.5(3)
Te(02)-O(005)#8	1.878(8)	O(005)#14-K(004)-O(005)#16	55.9(3)
Te(02)-O(005)	1.878(8)	O(005)#15-K(004)-O(005)#16	113.39(16)
Sc(03)-O(1)#12	2.070(10)	O(005)#14-K(004)-O(005)#17	113.39(16)
Sc(03)-O(1)#13	2.070(10)	O(005)#15-K(004)-O(005)#17	149.6(4)
Sc(03)-O(1)	2.070(10)	O(005)#16-K(004)-O(005)#17	59.5(3)
Sc(03)-O(005)#12	2.109(8)	O(005)#14-K(004)-O(005)#18	113.39(16)
Sc(03)-O(005)#13	2.109(8)	O(005)#15-K(004)-O(005)#18	55.9(3)
Sc(03)-O(005)	2.109(8)	O(005)#16-K(004)-O(005)#18	149.6(4)
K(004)-O(005)#14	3.005(3)	O(005)#17-K(004)-O(005)#18	113.39(16)
K(004)-O(005)#15	3.005(3)	O(005)#14-K(004)-O(005)#19	149.6(4)
K(004)-O(005)#16	3.005(3)	O(005)#15-K(004)-O(005)#19	113.39(16)
K(004)-O(005)#17	3.005(3)	O(005)#16-K(004)-O(005)#19	113.39(16)
K(004)-O(005)#18	3.005(3)	O(005)#17-K(004)-O(005)#19	55.9(3)
K(004)-O(005)#19	3.005(3)	O(005)#18-K(004)-O(005)#19	59.5(3)
K(004)-O(1)	3.125(15)	O(005)#14-K(004)-O(1)	76.4(2)
K(004)-O(1)#12	3.125(15)	O(005)#15-K(004)-O(1)	76.4(2)
K(004)-O(1)#13	3.125(15)	O(005)#16-K(004)-O(1)	102.13(17)
O(1)#1-Te(01)-O(1)#2	99.6(6)	O(005)#17-K(004)-O(1)	133.0(3)
O(1)#1-Te(01)-O(1)#3	99.6(6)	O(005)#18-K(004)-O(1)	102.13(18)
O(1)#2-Te(01)-O(1)#3	99.6(6)	O(005)#19-K(004)-O(1)	133.0(3)
O(005)#7-Te(02)-O(005)#8	97.1(3)	O(005)#14-K(004)-O(1)#12	102.13(17)
O(005)#7-Te(02)-O(005)	97.1(3)	O(005)#15-K(004)-O(1)#12	133.0(3)
O(005)#8-Te(02)-O(005)	97.1(3)	O(005)#16-K(004)-O(1)#12	76.4(2)
O(1)#12-Sc(03)-O(1)#13	91.9(5)	O(005)#17-K(004)-O(1)#12	76.4(2)
O(1)#12-Sc(03)-O(1)	91.9(5)	O(005)#18-K(004)-O(1)#12	133.0(3)
O(1)#13-Sc(03)-O(1)	91.9(5)	O(005)#19-K(004)-O(1)#12	102.13(18)
O(1)#12-Sc(03)-O(005)#12	89.0(3)	O(1)-K(004)-O(1)#12	56.8(3)
O(1)#13-Sc(03)-O(005)#12	178.7(5)	O(005)#14-K(004)-O(1)#13	133.0(3)
O(1)-Sc(03)-O(005)#12	89.0(3)	O(005)#15-K(004)-O(1)#13	102.13(18)
O(1)#12-Sc(03)-O(005)#13	89.0(3)	O(005)#16-K(004)-O(1)#13	133.0(3)
O(1)#13-Sc(03)-O(005)#13	89.0(3)	O(005)#17-K(004)-O(1)#13	102.13(17)
O(1)-Sc(03)-O(005)#13	178.7(5)	O(005)#18-K(004)-O(1)#13	76.4(2)
O(005)#12-Sc(03)-O(005)#13	90.0(3)	O(005)#19-K(004)-O(1)#13	76.4(2)
O(1)#12-Sc(03)-O(005)	178.7(5)	O(1)-K(004)-O(1)#13	56.8(3)
O(1)#13-Sc(03)-O(005)	89.0(3)	O(1)#12-K(004)-O(1)#13	56.8(3)

Symmetry transformations used to generate equivalent atoms:

#1 y-1,-x+y,z-1/2 #2 -x+1,-y+2,z-1/2 #3 x-y+1,x,z-1/2

#4 -x+1,-y+1,z-1/2 #5 -x,-y+1,z-1/2 #6 x,y,z-1

#7 -y+2,x-y+1,z #8 -x+y+1,-x+2,z #9 x+1,y+1,z

#10 x+1,y,z #11 -x+2,-y+2,z-1/2 #12 -y+1,x-y+1,z

#13 -x+y,-x+1,z #14 -x+1,-y+2,z+1/2 #15 y,-x+y+1,z+1/2

#16 x-y,x,z+1/2 #17 y-1,-x+y,z+1/2 #18 x-y+1,x,z+1/2

#19 -x+1,-y+1,z+1/2 #20 x-1,y,z #21 x-1,y-1,z

#22 -x+2,-y+2,z+1/2

Table S2 Selected bond distances (Å) and angles (deg) for RSTO.

Bond	Angle	Bond	Angle
Rb(4)-O(2)#1	3.0488(19)	O(2)#6-Rb(4)-O(008)	78.00(14)
Rb(4)-O(2)#2	3.0488(19)	O(008)#7-Rb(4)-O(008)	56.67(17)
Rb(4)-O(2)#3	3.0488(19)	O(008)#8-Rb(4)-O(008)	56.67(17)
Rb(4)-O(2)#4	3.0488(19)	O(2)#1-Rb(4)-O(008)#1	51.20(12)
Rb(4)-O(2)#5	3.0488(19)	O(2)#2-Rb(4)-O(008)#1	74.57(12)
Rb(4)-O(2)#6	3.0488(19)	O(2)#3-Rb(4)-O(008)#1	51.20(12)
Rb(4)-O(008)#7	3.167(8)	O(2)#4-Rb(4)-O(008)#1	96.87(16)
Rb(4)-O(008)#8	3.167(8)	O(2)#5-Rb(4)-O(008)#1	74.57(12)
Rb(4)-O(008)	3.167(8)	O(2)#6-Rb(4)-O(008)#1	96.87(16)
Rb(4)-O(008)#1	3.631(8)	O(008)#7-Rb(4)-O(008)#1	150.49(4)
Rb(4)-O(008)#4	3.631(8)	O(008)#8-Rb(4)-O(008)#1	120.02(2)
Rb(4)-O(008)#5	3.631(8)	O(008)-Rb(4)-O(008)#1	150.49(4)
Te(1)-O(2)#9	1.875(6)	O(2)#1-Rb(4)-O(008)#4	74.57(12)
Te(1)-O(2)#10	1.875(6)	O(2)#2-Rb(4)-O(008)#4	51.20(12)
Te(1)-O(2)	1.875(6)	O(2)#3-Rb(4)-O(008)#4	96.87(16)
Te(2)-O(008)#13	1.851(6)	O(2)#4-Rb(4)-O(008)#4	51.20(12)
Te(2)-O(008)#14	1.851(6)	O(2)#5-Rb(4)-O(008)#4	96.87(16)
Te(2)-O(008)	1.851(6)	O(2)#6-Rb(4)-O(008)#4	74.57(12)
Sc(3)-O(008)	2.089(6)	O(008)#7-Rb(4)-O(008)#4	120.02(2)
Sc(3)-O(008)#8	2.089(6)	O(008)#8-Rb(4)-O(008)#4	150.49(4)
Sc(3)-O(008)#7	2.089(6)	O(008)-Rb(4)-O(008)#4	150.49(4)
Sc(3)-O(2)#8	2.115(6)	O(008)#1-Rb(4)-O(008)#4	45.88(15)
Sc(3)-O(2)#7	2.115(6)	O(2)#1-Rb(4)-O(008)#5	96.87(16)
Sc(3)-O(2)	2.115(6)	O(2)#2-Rb(4)-O(008)#5	96.87(16)
O(2)#1-Rb(4)-O(2)#2	55.1(2)	O(2)#3-Rb(4)-O(008)#5	74.57(12)
O(2)#1-Rb(4)-O(2)#3	59.3(2)	O(2)#4-Rb(4)-O(008)#5	74.57(12)
O(2)#2-Rb(4)-O(2)#3	112.03(11)	O(2)#5-Rb(4)-O(008)#5	51.20(12)
O(2)#1-Rb(4)-O(2)#4	112.03(11)	O(2)#6-Rb(4)-O(008)#5	51.20(12)
O(2)#2-Rb(4)-O(2)#4	59.3(2)	O(008)#7-Rb(4)-O(008)#5	150.49(4)
O(2)#3-Rb(4)-O(2)#4	146.4(2)	O(008)#8-Rb(4)-O(008)#5	150.49(4)
O(2)#1-Rb(4)-O(2)#5	112.03(11)	O(008)-Rb(4)-O(008)#5	120.02(2)
O(2)#2-Rb(4)-O(2)#5	146.4(2)	O(008)#1-Rb(4)-O(008)#5	45.88(15)
O(2)#3-Rb(4)-O(2)#5	55.1(2)	O(008)#4-Rb(4)-O(008)#5	45.88(15)
O(2)#4-Rb(4)-O(2)#5	112.03(11)	O(2)#9-Te(1)-O(2)#10	97.6(2)
O(2)#1-Rb(4)-O(2)#6	146.4(2)	O(2)#9-Te(1)-O(2)	97.6(2)
O(2)#2-Rb(4)-O(2)#6	112.03(11)	O(2)#10-Te(1)-O(2)	97.6(2)
O(2)#3-Rb(4)-O(2)#6	112.03(11)	O(008)#13-Te(2)-O(008)#14	99.7(3)
O(2)#4-Rb(4)-O(2)#6	55.1(2)	O(008)#13-Te(2)-O(008)	99.7(3)
O(2)#5-Rb(4)-O(2)#6	59.3(2)	O(008)#14-Te(2)-O(008)	99.7(3)
O(2)#1-Rb(4)O(008)#7	103.37(11)	O(008)#13-Te(2)-Rb(4)#18	127.57(6)

O(2)#2-Rb(4)O(008)#7	78.00(14)	O(008)#14-Te(2)-Rb(4)#18	55.9(2)
O(2)#3-Rb(4)O(008)#7	134.49(15)	O(008)-Sc(3)-O(008)#8	92.0(3)
O(2)#4-Rb(4)-O(008)#7	78.00(14)	O(008)-Sc(3)-O(008)#7	92.0(3)
O(2)#5-Rb(4)-O(008)#7	134.49(15)	O(008)#8-Sc(3)-O(008)#7	92.0(3)
O(2)#6-Rb(4)-O(008)#7	103.37(11)	O(008)-Sc(3)-O(2)#8	88.49(16)
O(2)#1-Rb(4)-O(008)#8	78.00(14)	O(008)#8-Sc(3)-O(2)#8	88.49(16)
O(2)#2-Rb(4)-O(008)#8	103.37(11)	O(008)#7-Sc(3)-O(2)#8	179.2(3)
O(2)#3-Rb(4)-O(008)#8	78.00(14)	O(008)-Sc(3)-O(2)#7	179.2(3)
O(2)#4-Rb(4)-O(008)#8	134.49(15)	O(008)#8-Sc(3)-O(2)#7	88.49(16)
O(2)#5-Rb(4)-O(008)#8	103.37(11)	O(008)#7-Sc(3)-O(2)#7	88.49(16)
O(2)#6-Rb(4)-O(008)#8	134.49(15)	O(2)#8-Sc(3)-O(2)#7	91.0(3)
O(008)#7-Rb(4)-O(008)#8	56.67(17)	O(008)-Sc(3)-O(2)	88.49(16)
O(2)#1-Rb(4)-O(008)	134.49(15)	O(008)#8-Sc(3)-O(2)	179.2(3)
O(2)#2-Rb(4)-O(008)	134.49(15)	O(008)#7-Sc(3)-O(2)	88.49(16)
O(2)#3-Rb(4)-O(008)	103.37(11)	O(2)#8-Sc(3)-O(2)	91.0(2)
O(2)#4-Rb(4)-O(008)	103.37(11)	O(2)#7-Sc(3)-O(2)	91.0(3)
O(2)#5-Rb(4)-O(008)	78.00(14)		

Symmetry transformations used to generate equivalent atoms:

#1 y,-x+y,z-1/2 #2 -x+2,-y+1,z-1/2 #3 x-y,x-1,z-1/2

#4 x-y+1,x,z-1/2 #5 -x+1,-y+1,z-1/2 #6 y,-x+y+1,z-1/2

#7 -x+y+1,-x+1,z #8 -y+1,x-y,z #9 -x+y+1,-x+2,z

#10 -y+2,x-y+1,z #11 x+1,y+1,z #12 x,y+1,z

#13 -y+1,x-y+1,z #14 -x+y,-x+1,z #15 -x+2,-y+2,z+1/2

#16 -x+1,-y+1,z+1/2 #17 -x+2,-y+1,z+1/2 #18 x-1,y,z

#19 x,y-1,z #20 x-1,y-1,z

Table S3 Atomic coordinates (× 10^4), equivalent isotropic displacement parameters (Å^2 × 10^3), and the bond-valence sum for KSTO. Ueq is defined as one third of the trace of the orthogonalized Uij tensor.

Atoms	x	y	z	U(eq)	BVS
Te(01)	3333	6667	1738(1)	11(1)	4.24
Te(02)	10000	10000	4743(1)	12(1)	3.92
Sc(03)	3333	6667	4983(3)	10(1)	3.14
K(004)	3333	6667	8246(6)	21(1)	0.77
O(1)	5043(10)	10090(20)	5984(12)	48(3)	2.03
O(005)	6763(14)	8381(7)	3928(7)	22(2)	1.90

Table S4 Atomic coordinates (× 10^4), equivalent isotropic displacement parameters (Å^2 × 10^3), and the bond-valence sum for RSTO. Ueq is defined as one third of the trace of the orthogonalized Uij tensor.

Atoms	x	y	z	U(eq)	BVS
Rb(4)	6667	3333	2392(2)	17(1)	0.97
Te(1)	10000	10000	5857(1)	10(1)	3.95
Te(2)	3333	6667	3902(1)	9(1)	4.22
Sc(3)	6667	3333	5627(2)	9(1)	3.03
O(2)	8389(6)	6779(13)	6646(5)	15(1)	1.92
O(008)	4950(6)	5050(6)	4640(6)	31(2)	2.01

Table S5. Comparison of UV cutoff edges between RSTO and other representative NLO tellurate crystals.

Compound	crystal system	space group	UV cutoff edge (μm)
$V_2Te_2O_9$	orthorhombic	*Fdd*2	0.62
$Cs_2Mo_3TeO_{12}$	hexagonal	$P6_3$	0.43
$Na_2Mo_3Te_3O_{16}$ [1]	monoclinic	*I*2	0.42
$Cs_2W_3TeO_{12}$ [2]	hexagonal	$P6_3$	0.41
$MnMoTeO_6$ [3]	orthorhombic	$P2_12_12_1$	0.41
Bi_2TeO_5 [4]	orthorhombic	*Abm*2	0.4
β-$BaMo_2TeO_9$	monoclinic	$P2_1$	0.4
α-$BaMo_2TeO_9$ [5]	orthorhombic	$Pca2_1$	0.38
$Mo(H_2O)Te_2O_7$ [6]	Tetragonal	$I\bar{4}$	0.366
$Na_2W_2TeO_9$ [7]	monoclinic	*Ia*	0.36
$MgMoTeO_6$ [8]	orthorhombic	$P2_12_12_1$	0.36
$ZnMoTeO_6$ [9]	orthorhombic	$P2_12_12_1$	0.35
$CdMoTeO_6$	tetragonal	$P\bar{4}2_1m$	0.35
$Nb_2Te_3O_{11}$ [10]	orthorhombic	$P2_12_12_1$	0.334
$Cd_2Nb_2Te_4O_{15}$ [11]	orthorhombic	$Pca2_1$	0.33
$LiNbTeO_5$ [12]	monoclinic	$P2_1$	0.33
β-BaW_2TeO_9 [13]	monoclinic	$P2_1$	0.325
Zn_2MoTeO_7 [14]	monoclinic	$P2_1$	0.3
$Cd_3WTe_2O_{10}$ [15]	monoclinic	$P2_1$	0.29
$Ca_3MoTe_2O_{10}$ [16]	monoclinic	$P2_1$	0.279
$Ca_7MoTe_6O_{22}$ [17]	monoclinic	*C*2	0.266
$RbScTe_2O_6$ this work	hexagonal	$P6_3mc$	0.236

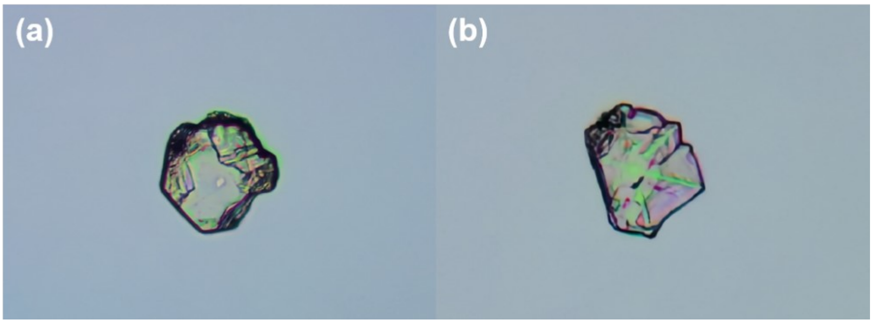

Fig. S1 Crystal morphology of (a) RSTO and (b) KSTO.

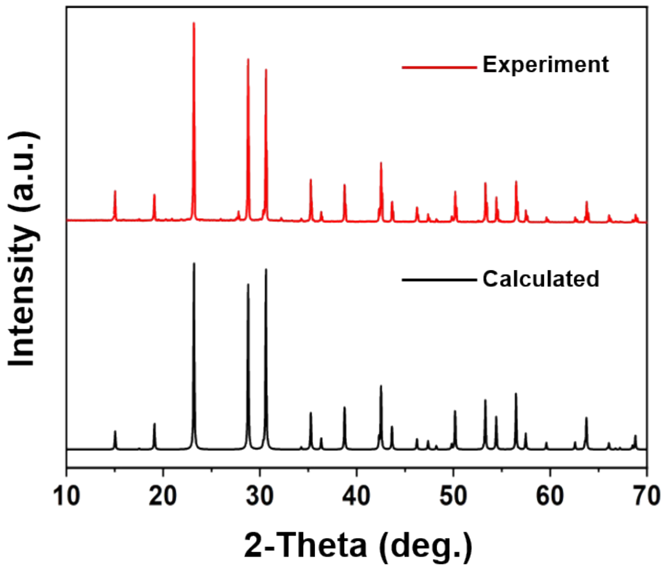

Fig. S2 Experimental and calculated PXRD patterns of RSTO.

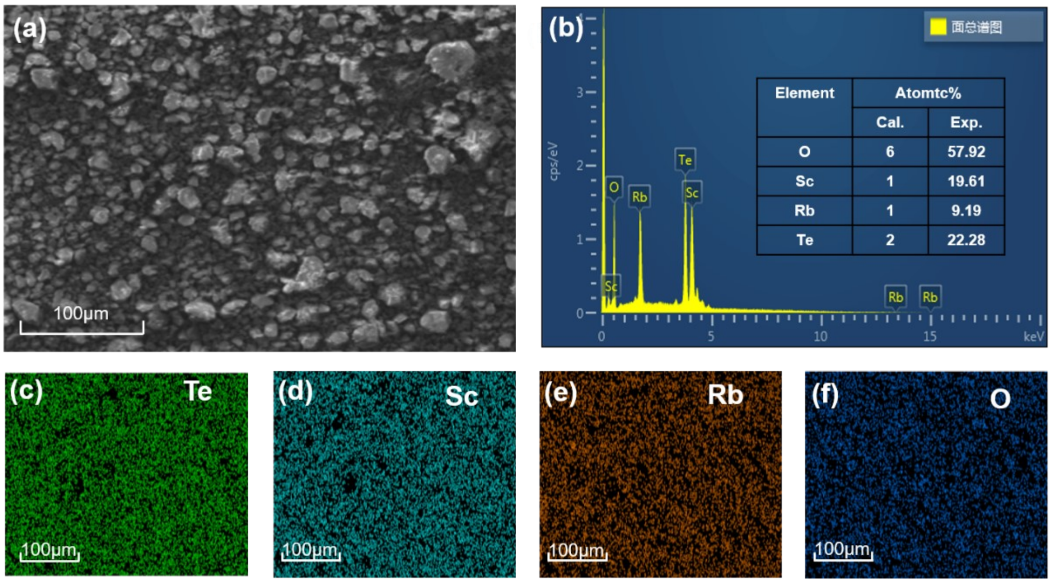

Fig. S3 EDS analysis of RSTO. (a) Surface morphology. (b) Atomic ratio of various elements. (c-f) Te, Sc, Rb, and O mapping results, respectively.

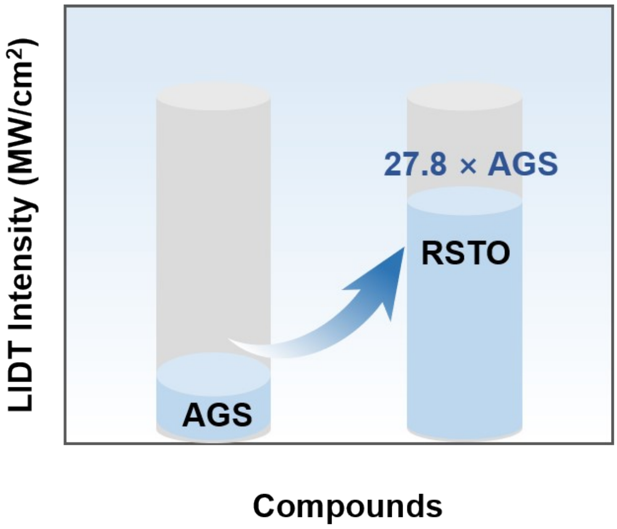

Fig. S4 Comparison of the LIDT values for AGS and RSTO.

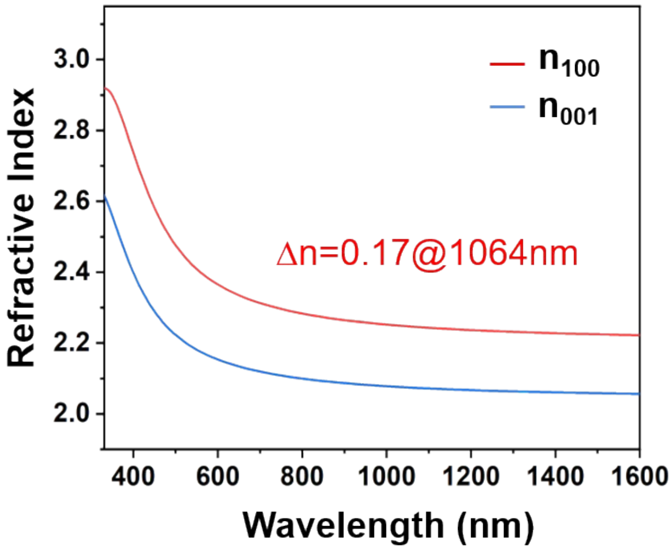

Fig. S5 Calculated refractive index dispersion curves of KSTO derived from DFT-based simulations.

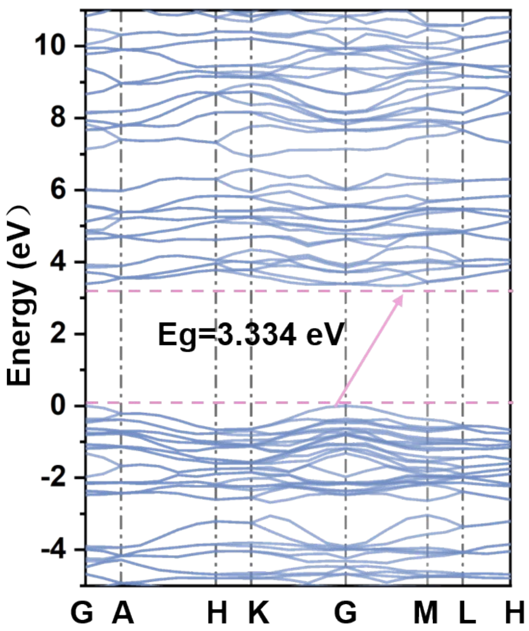

Fig. S6 Calculated band structure of KSTO.

REFERENCES

1 W. L. Zhang, J. F. Sun, X. Q. Wang, G. Q. Shen and D. Z. Shen, *CrystEngComm*, 2012, **14**, 3490-3494.

2 J. J. Zhang, X. T. Tao, Y. X. Sun, Z. H. Zhang, C. Q. Zhang, Z. L. Gao, H. B. Xia and S. Q. Xia, *Cryst. Growth Des.*, 2011**,** **11**, 1863-1868.

3 C. G. Jin, Z. Li, L. X. Huang and M. Z. He, *J. Cryst. Growth.*,2013, **369**, 43-46.

4 I. Földvári, A. Peter and R. Voszka, *J. Cryst. Growth.*, 1990, **100**, 75-77.

5 J. J. Zhang, Z. H. Zhang, Y. X. Sun, C. Q. Zhang and T. X. Tao, *CrystEngComm*, 2011, **13,** 6985-6990.

6 T. H. Wu, X. X. Jiang, K. Duanmu and C. Wu, *Adv. Sci.*, 2024, **11**, 2306670.

7 W. G. Zhang, F. Li, S.-H Kim and P. S.Halasyamani, *Cryst. Growth Des.*, 2010, **10**, 4091-4095.

8 J. J. Zhang, Z. H. Zhang, Y. X. Sun, C. Q. Zhang, S. J. Zhang, Y. and X. T. Tao, *J. Mater. Chem.*, 2012, **22,** 9921-9927.

9 S. G. Zhao, J. H. Luo, P. Zhou, S. Q. Zhang, Z. H. Sun and M. C. Hong, *RSC Adv.*, 2013, **3**, 14000-14006.

10 T. H. Zhang, J. J. Jiao, W. L. Zhao, F. Wang, F. Liang, N. Ye, Z. G. Hu, Y. C. Wu and C. G. Li, *Inorg. Chem.*, 2023, **62**, 17522-17529.

11 Q. Wang, X. H. Dong, L. Huang, K. M. Ok, Z. E. Lin and G. H. Zou, *Small*, 2023, **19**, e2302797.

12 K. C. Chen, C. S. Lin, G. Peng, Y. Chen, H. Z. Huang, E. Z. Chen, Y. X. Min, T. Yan, M. Luo and N. Ye, *Chem. Mater.*, 2022, **34**, 399-404.

13 Z. Z. Zhang, X. T. Tao, J. J. Zhang, Y. X. Sun, C. Z. Zhang and B. Li, *CrystEngComm*, 2013, **15**, 10197-10204.

14 W. G. Zhang and P. S. Halasyamani, *J. Solid State Chem.*, 2016, **236**, 32-38.

15 Q. X. Zhang, Q. Wu, J. F. Zhou, C. Tang and M. J. Xia, *Cryst. Growth Des.*, 2022, **22**, 6678-6685.

16 Q. Wu, J. F. Zhou, X. L. Liu, X. X. Jiang., Q. X. Zhang, Z. S. Lin and M. J. Xia， *Inorg. Chem. Front.,* 2021, **60**, 18512-18520.

17 T. H. Zhang, F. Liang, C. G. Li, N. Ye, and Z. G. Hu, *J. Am. Chem. Soc.,* 2025, **147**, 18275-18283.
